# Genome-Wide Association Mapping of Hulless Barely Phenotypes in Drought Environment

**DOI:** 10.3389/fpls.2022.924892

**Published:** 2022-06-23

**Authors:** Jie Li, Xiaohua Yao, Youhua Yao, Likun An, Zongyun Feng, Kunlun Wu

**Affiliations:** ^1^College of Agronomy Sichuan Agricultural University, Chengdu, China; ^2^Academy of Agricultural and Forestry Sciences, Qinghai University, Xining, China; ^3^Qinghai Key Laboratory of Hulless Barley Genetics and Breeding, Xining, China; ^4^Qinghai Subcenter of National Hulless Barley Improvement, Xining, China; ^5^State Key Laboratory of Crop Gene Exploration and Utilization in Southwest China, Chengdu, China

**Keywords:** hulless barley, GWAS, drought resistance, high throughput sequencing, quantitative traits, SNP

## Abstract

Drought stress is one of the main factors restricting hulless barley (*Hordeum vulgare* L. var. *nudum* Hook. f.) yield. Genome-wide association study was performed using 269 lines of hulless barley to identify single-nucleotide polymorphism (SNP) markers associated with drought-resistance traits. The plants were cultured under either normal or drought conditions, and various quantitative traits including shoot fresh weight, shoot dry weight, root fresh weight, root dry weight, leaf fresh weight, leaf saturated fresh weight, leaf dry weight, ratio of root and shoot fresh weight, ratio of root and shoot dry weight, shoot water loss rate, root water loss rate, leaf water content and leaf relative water content, and field phenotypes including main spike length, grain number per plant, grain weight per plant, thousand grain weight (TGW), main spike number, plant height, and effective spike number of plants were collected. After genotyping the plants, a total of 8,936,130 highly consistent population SNP markers were obtained with integrity > 0.5 and minor allele frequency > 0.05. Eight candidate genes potentially contributed to the hulless barley drought resistance were obtained at loci near significant SNPs. For example, *EMB506, DCR*, and *APD2* genes for effective spike number of plants, *ABCG11* gene for main spike number (MEN), *CLPR2* gene for main spike length, *YIP4B* gene for root and shoot dry weight (RSWD), and *GLYK* and *BTS* genes for TGW. The SNPs and candidate genes identified in this study will be useful in hulless barley breeding under drought resistance.

## Introduction

Plants live in complex and changeable environmental conditions, often bring huge misfortune on plant growth (Zhu, 2016). As the global climate becomes drier and warmer, more than 15% of the world's population faces severe water shortages (Schewe et al., 2014; Gong et al., 2020). Drylands cover 40% of the global land surface and drought has caused losses in agriculture up to $30 billion over the past decade (Dai, 2013; Gupta et al., 2020). Drought has brought a great strain on the growth of plants, at the meantime, plants also have corresponding effective measures to prevent water loss, maintain cell water content, and help plants to survive the difficult drought period. Understanding drought resistance and water use efficiency of plants will provide guarantee for maintaining normal plant growth and improving agricultural yield under drought (Gupta et al., 2020; Yu et al., 2021).

Hulless barley (*Hordeum vulgare* L. var. *nudum* Hook. f.) is an important economic crop (He and Jia, 2008). As the only crop growing at high altitude, the planting area of hulless barley accounts for 43% of the grain crop area on the Qinghai Tibet Plateau (Dai et al., 2012; Zhong et al., 2016). Hulless barley has made great contribution as the main food, fuel, and livestock feed of the Tibetan people, and also is the raw material for beer, medicine, and health care products (Yang et al., 2013; Zhu et al., 2015; Liu et al., 2018). Hulless barley is rich in β-glucan, phenolic acid, and anthocyanins, which has high nutritional and medicinal value and is of great significance to human health (Bonoli et al., 2004; Siebenhandl et al., 2007; Kohyama et al., 2008; Zhao et al., 2015). The climate inside the Qinghai–Tibet Plateau is gradually drying out, and some scientists predict that only plants that can tolerate drought conditions will be able to settle on the plateau's platforms (Meng et al., 2017). Therefore, it is very important to study the drought tolerance of hulless barley.

To predict the important agronomic traits such as drought tolerance, it is necessary to understand the specific loci based on phenotype and the genetic structure of the traits. Genome-wide association study (GWAS) is just such a powerful tool for connecting genotypes–phenotypes (Korte and Farlow, 2013). Genome-wide association study refers to the association analysis of traits through the sequence and the SNP marker information on the whole genome so as to detect the loci significantly associated with the target trait (Li, 2013; Tam et al., 2019). Genome-wide association study provides higher resolution and finer scale association, and has been widely used in the identification of markers associated with desirable traits in crops (Nordborg and Weigel, 2008; Xu et al., 2017).

This study based on the identification results of hulless barley drought tolerance traits in 269 lines, SNP markers were developed by simplified genome sequencing (SLAF) to genotype natural populations. Using linear mixed model (LMM) and EmMax, the association between the quantitative traits of drought tolerance and genotype was analyzed, and the SNP loci and chromosome segments significantly associated with the target traits were screened.

## Materials and Methods

### Genetic Materials

The 269 hulless barley lines with different drought resistance assessment were used as the GWAS panel in this study (Supplementary Table 1). Phenotypic observation was performed on each line, both in the laboratory and in the field. The laboratory experiment was conducted in two growth condition with three biological replicates. The normal culture group was used as control, and the treatment group was applied with PEG-6000 to simulate drought stress. The associated phenotypes including shoot fresh weight SFW (g),shoot dry weight SDW (g), root fresh weight RFW (g), root dry weight RDW (g), leaf fresh weight LFW (g), leaf saturated fresh weight SFW (g), leaf dry weight LDW (g), ratio of root and shoot fresh weight RSFW (%), ratio of root and shoot dry weight RSWD (%), shoot water loss rate SWLR (%), root water loss rate RWLR (%), leaf water content WC (%), and leaf relative water content RWC (%) were measured. Field planting data were collected in 2019–2020 from three different growing environments at two sites, including drought treatment and natural irrigation at two different habitats. The associated phenotypes of different habitats consisted of main spike length MSL (cm), grain number per plant GNPP, grain weight per plant GWPP (g), thousand grain weight TGW(g), main spike number MEN(g), plant height (cm) and effective spike number of plants ESNP.

### Single-Nucleotide Polymorphism-Based Genotyping for 269 Hulless Barley Lines

In 2021, 269 pieces of hulless barley lines were planted in germinating boxes and cultured in greenhouse to two leaves stage. Whole-genome DNA of each germplasm resource leaves was extracted by CTAB method (Allen et al., 2006). The DNA quality and concentration were detected by 0.1% agarose gel electrophoresis, and whole-genome SNP genotyping was produced by Biomarker technologies company. The SLAF tags were developed by enzyme digestion (RsaI) of the genomic DNA, followed by adaptor ligation, amplification and purification. Then, the SLAF library were sequenced by Illumina Novaseq 6000. The sequencing reads were mapped to the reference genome by BWA software (Li and Durbin, 2009). GATK (McKenna et al., 2010) and samtools (Li et al., 2009) were used to identify SNPs. The intersection of SNP markers obtained by the two methods was used as the final reliable SNP marker dataset, and a total of 5,949,446 SNPs were obtained. The genotypic data obtained were screened as integrity > 0.8 and minor allele frequency (MAF) > 0.05.

### Structure of Hulless Barley Population

Based on the SNPs obtained from the above genotypes, 269 phylogenetic trees of hulless barley was constructed by neighbor-joining (NJ) method (1,000 replicates) with Kimura 2-parameter (K2-P) model using MEGA X software (Kumar et al., 2018). The phylogenetic tree was colored based on the analysis results of STRUCTURE.

### Genome-Wide Association Study and Candidate Gene Screening

Based on the developed high-density SNP molecular markers, GEMMA, FaST-LMM, and EMMAX were used for association analysis. Correlation analysis between phenotypic value of drought-tolerant-resistant traits and genotypes was carried out to obtain the *p*-value of each SNP. Screened with *p* < 5 × 10^−6^, the genetic variation loci most likely to affect the trait was selected. The quantile–quantile (Q–Q) scatter plot and Manhattan plot were made by the qqman package in R software.

To screen the drought-tolerant-resistant genes near the significant associative loci, the genetic information of specific association regions was queried from barley genome in plant whole-genome information database (http://plants.ensembl.org/index.html). All genes with coding regions in the 100–500-kb window were used for subsequent analysis.

## Results

### Genomic Library Construction and SNP Markers Development

**Figure 1:**
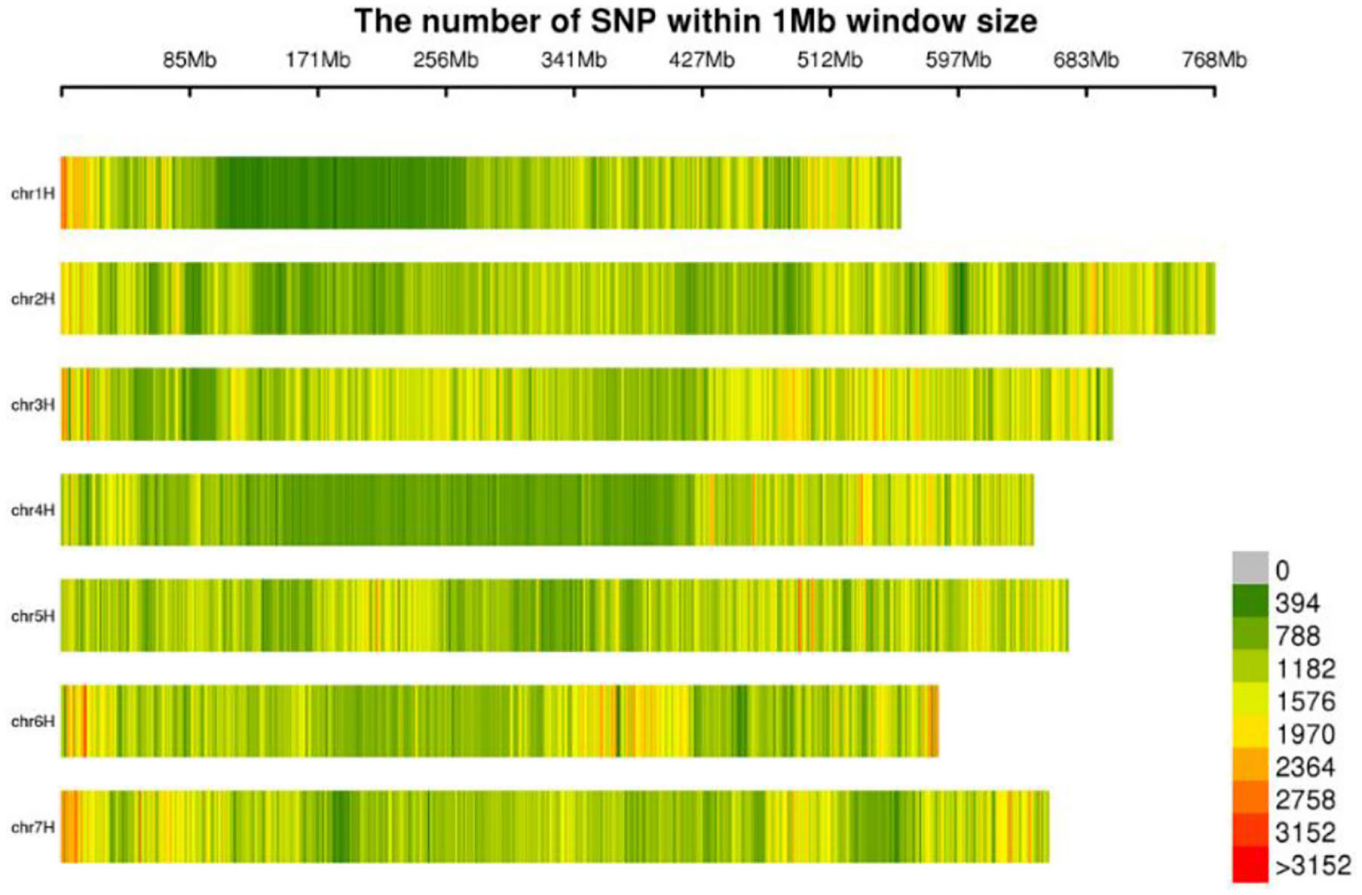
Distribution of SNPs on chromosomes.

The molecular markers of 269 hulless barley lines were developed by Specific-Locus Amplified Fragment Sequencing (LAF-SEQ) to obtain molecular markers in the whole genome. An average of 311,695 SLAF tags were developed per sample for a total of 862,999, including 480,790 polymorphic SLAF tags and 5,532,468 SNP markers. The average sequencing depth of SLAF tags was 10.43 ×, and 1,067.96 Mb reads data were generated. These markers were evenly distributed on the chromosomes of hulless barley (Table 1, Figure 1). The average Q30 of sequences was 94.78%, and the average GC content was 44.23%. A total of 8,936,130 SNP markers with high consistency were obtained from 269 hulless barley lines filtered by integrity > 0.5 and MAF > 0.05. Chromosome 3 had the largest number of SNP markers (1,532,190), with an average label distance of 456 bp. On the contrary, chromosome 1 had the lowest number of SNP markers (914,610), with an average label distance of 610 bp.

**Table 1:** Distribution of SLAF markers on chromosomes.

**Chromosome ID**	**Chromosome length**	**SNP number**	**SNP number**	**Polymorphic SLAF**
chr1H	558,535,432	914,610	103,229	55,541
chr2H	768,075,024	1,289,234	139,589	77,829
chr3H	699,711,114	1,532,190	130,401	75,513
chr4H	647,060,158	1,186,220	122,917	67,774
chr5H	670,030,160	1,451,138	117,223	66,198
chr6H	583,380,513	1,216,744	108,416	63,442
chr7H	657,224,000	1,345,994	118,728	68,117
chrUn	249,774,706	123,320	22,256	6,334

### Genetic Structure of Hulless Barley Population

**Figure 2:**
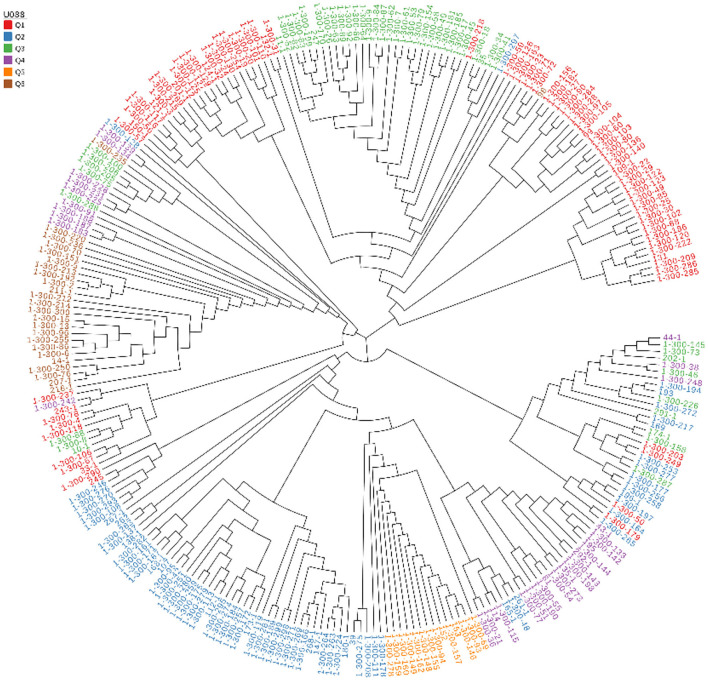
Genetic relatedness among the 269 hulless barley lines estimated by neighbor-joining method and represented as a polar tree diagram. The estimated genetic relatedness is based on 5,949,446 SNPs identified by genotyping-by-sequencing and filtered for MAF of 0.05.

The 269 lines were divided into 6 groups according to the geographic location information of the samples, and the group information was used for linkage disequilibrium (LD) and evolutionary tree analysis. The phylogenetic tree was colored by the clustering result of STRUCTURE, basically, each cluster was gathered into one block in the phylogenetic tree, especially Q5 and Q6 (Figure 2).

**Figure 3:**
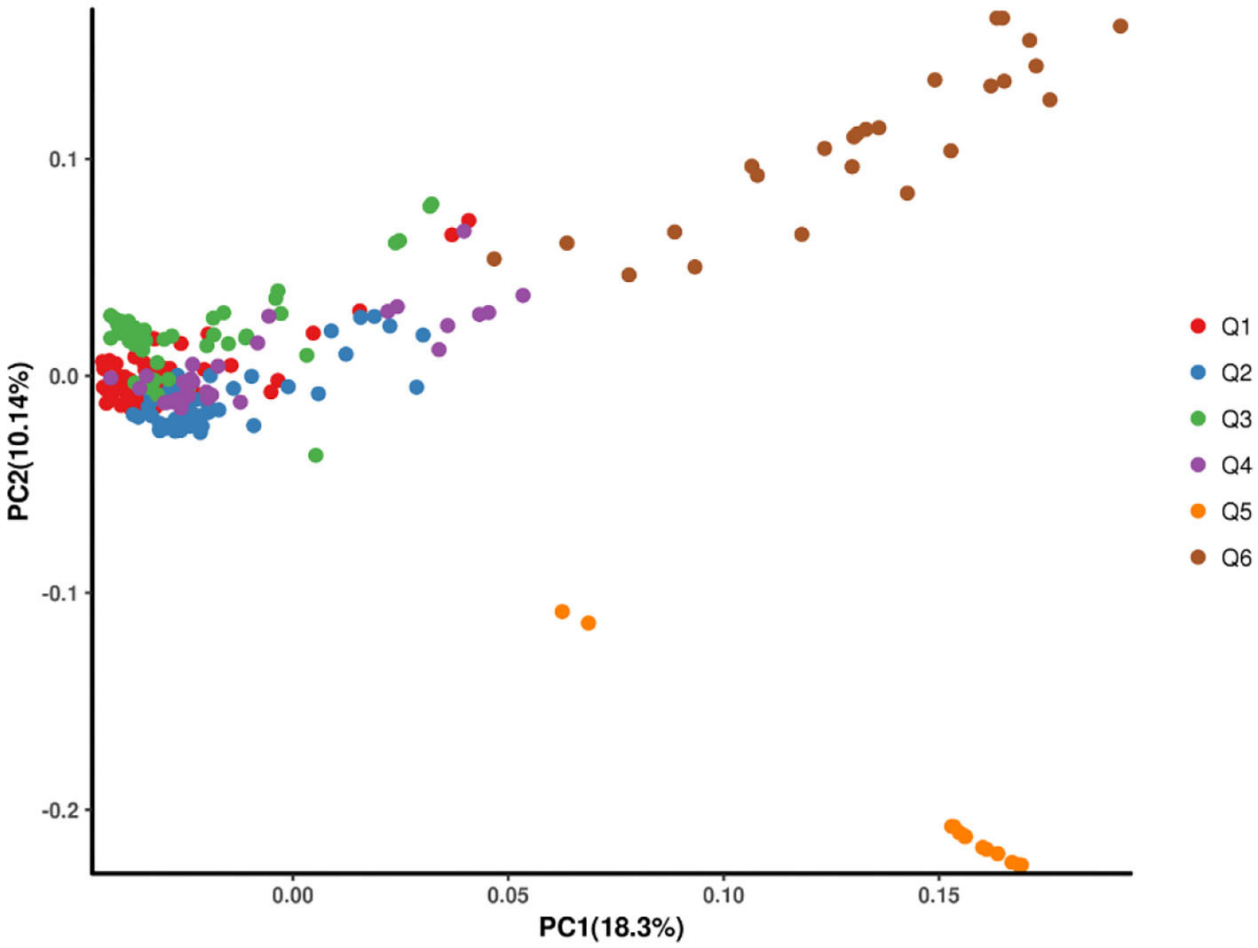
The scatter plots of the first two principal components (PCs) showing the distribution of the 269 hulless barley lines in PC1 vs. PC2.

Using the SNP information mentioned above, the principal component analysis (PCA) was conducted. The top three principal components could explain 34.23% of the genomic variations, and principal component 1 could explain 18.3%. Consistent with the phylogenetic tree, Q5 and Q6 were separate from other populations (Figure 3).

**Figure 4:**
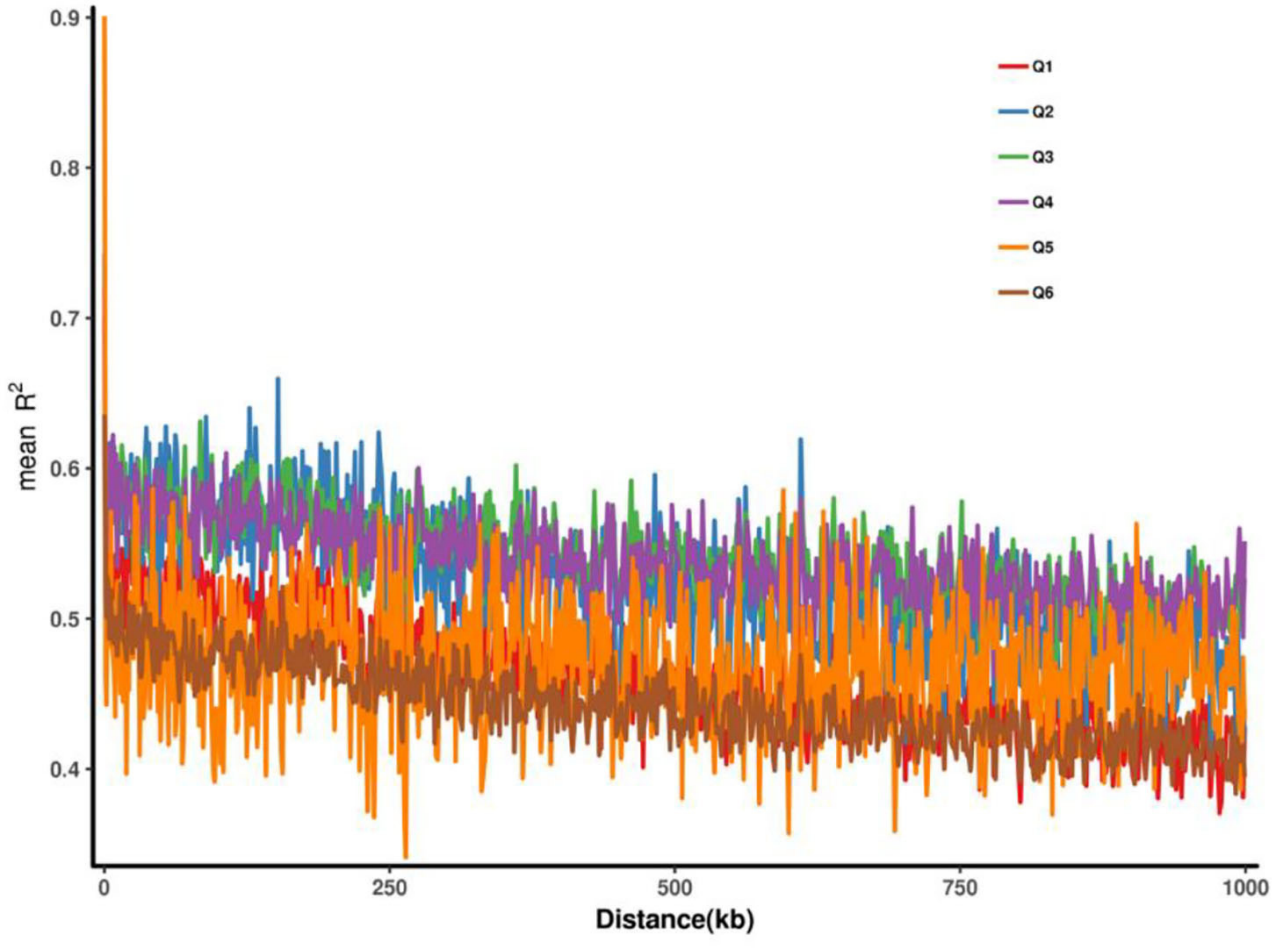
Linkage disequilibrium decay based on six groups.

Plink2 software (Chen et al., 2019) was used to calculate the linkage disequilibrium (LD) between two SNP pairs within a certain distance (1,000 kb) on the same chromosome, and the linkage disequilibrium intensity was represented by *r*^2^. The closer *r*^2^ is to 1, the stronger the linkage disequilibrium intensity. The distance between SNPs and *r*^2^ was fitted, and the curve of *r*^2^ changes with distance was presented. Generally speaking, the closer the distance between SNPs is, the larger *r*^2^ is and *vice versa*. The LD decay (LDD) distance was used as the distance traveled when the maximum *r*^2^ value dropped to half. The longer LDD, the lower the probability of recombination within the same physical distance. It should be noted that some regions of Q5 and Q6 had very strong linkage, but the length of the strong linkage was short, indicating that these two groups were subjected to some artificial selection pressure and some loci were selected, leading to linkage in some regions (Figure 4).

### Genome-Wide Association Study Analysis of Traits in Hulless Barley

This analysis was based on SNP data from mutation detection, filtered by secondary allele frequency (MAF > 0.05) and locus integrity (integrity > 0.8) to obtain highly consistent SNP loci for GWAS analysis. Genome-wide association analysis was performed using LMM, EMMAX, and FaST-LMM models, respectively. The following table showed the number of significant SNP markers obtained for each phenotype corresponding to each mode and the number of common SNP markers in each model (Table 2).

**Table 2:** SNP markers for each phenotype based on different GWAS analysis models.

**Phenotype**	**Year**	**Station**	**Conditions**	**LMM**	**EMMAX**	**FASTLMM**	**Shared SNP markers**
**The field data**							
ESN	2019	Menyuan	Field	18	6	10	6
	2019	Xining	Field	84	22	54	21
	2019	Xining	Greenhouse	10	3	7	3
	2020	Menyuan	Field	44	10	18	10
	2020	Xining	Field	117	6	74	6
	2020	Xining	Greenhouse	5	1	2	1
GW	2019	Menyuan	Field	10	3	11	3
	2019	Xining	Field	11	0	14	0
	2019	Xining	Greenhouse	6	1	6	1
	2020	Menyuan	Field	4	1	1	1
	2020	Xining	Field	11	1	6	1
	2020	Xining	Greenhouse	0	0	0	0
MSL	2019	Menyuan	Field	2	0	2	0
	2019	Xining	Field	7	2	5	2
	2019	Xining	Greenhouse	1	0	1	0
	2020	Menyuan	Field	191	1	41	1
	2020	Xining	Field	6	0	1	0
	2020	Xining	Greenhouse	1	0	2	0
PH	2019	Menyuan	Field	10	2	39	2
	2019	Xining	Field	7	1	6	1
	2019	Xining	Greenhouse	28	10	23	8
	2020	Menyuan	Field	14	2	6	2
	2020	Xining	Field	2	0	3	0
	2020	Xining	Greenhouse	0	0	0	0
SN	2019	Menyuan	Field	37	2	108	1
	2019	Xining	Field	32	26	34	23
	2019	Xining	Greenhouse	2	1	2	1
	2020	Menyuan	Field	24	6	79	6
	2020	Xining	Field	3	3	3	3
	2020	Xining	Greenhouse	5	3	5	3
SPP	2019	Menyuan	Field	0	0	0	0
	2019	Xining	Field	28	0	13	0
	2019	Xining	Greenhouse	12	1	7	1
	2020	Menyuan	Field	0	0	4	0
	2020	Xining	Field	7	0	3	0
	2020	Xining	Greenhouse	1	1	1	1
TGW	2019	Menyuan	Field	16	17	24	16
	2019	Xining	Field	14	2	8	2
	2019	Xining	Greenhouse	15	1	2	1
	2020	Menyuan	Field	47	33	55	31
	2020	Xining	Field	25	1	19	1
	2020	Xining	Greenhouse	4	0	4	0
**Laboratory data**							
SFW			Control	3	3	3	3
			PEG	2	0	1	0
LDMC			Control	0	0	0	0
			PEG-6000	0	0	0	0
LDW			Control	4	0	5	0
			PEG-6000	5	1	1	1
LFW			Control	3	0	0	0
			PEG-6000	2	0	2	0
RDW			Control	1	0	0	0
			PEG-6000	2	1	2	1
RFW			Control	0	0	0	0
			PEG-6000	13	3	10	3
RSDW			Control	3	0	3	0
			PEG-6000	3	4	5	2
RSFW			Control	0	0	0	0
			PEG-6000	3	2	2	2
RWC			Control	1	1	1	1
			PEG-6000	23	1	48	1
RWLR			Control	3	3	4	2
			PEG-6000	1	2	2	1
SDW			Control	5	0	5	0
			PEG-6000	2	1	2	1
SWLR			Control	1,148	0	696	0
			PEG-6000	1	2	2	1
WC			Control	1	1	1	1
			PEG-6000	10	11	14	7

### Association Analysis

**Figure 5:**
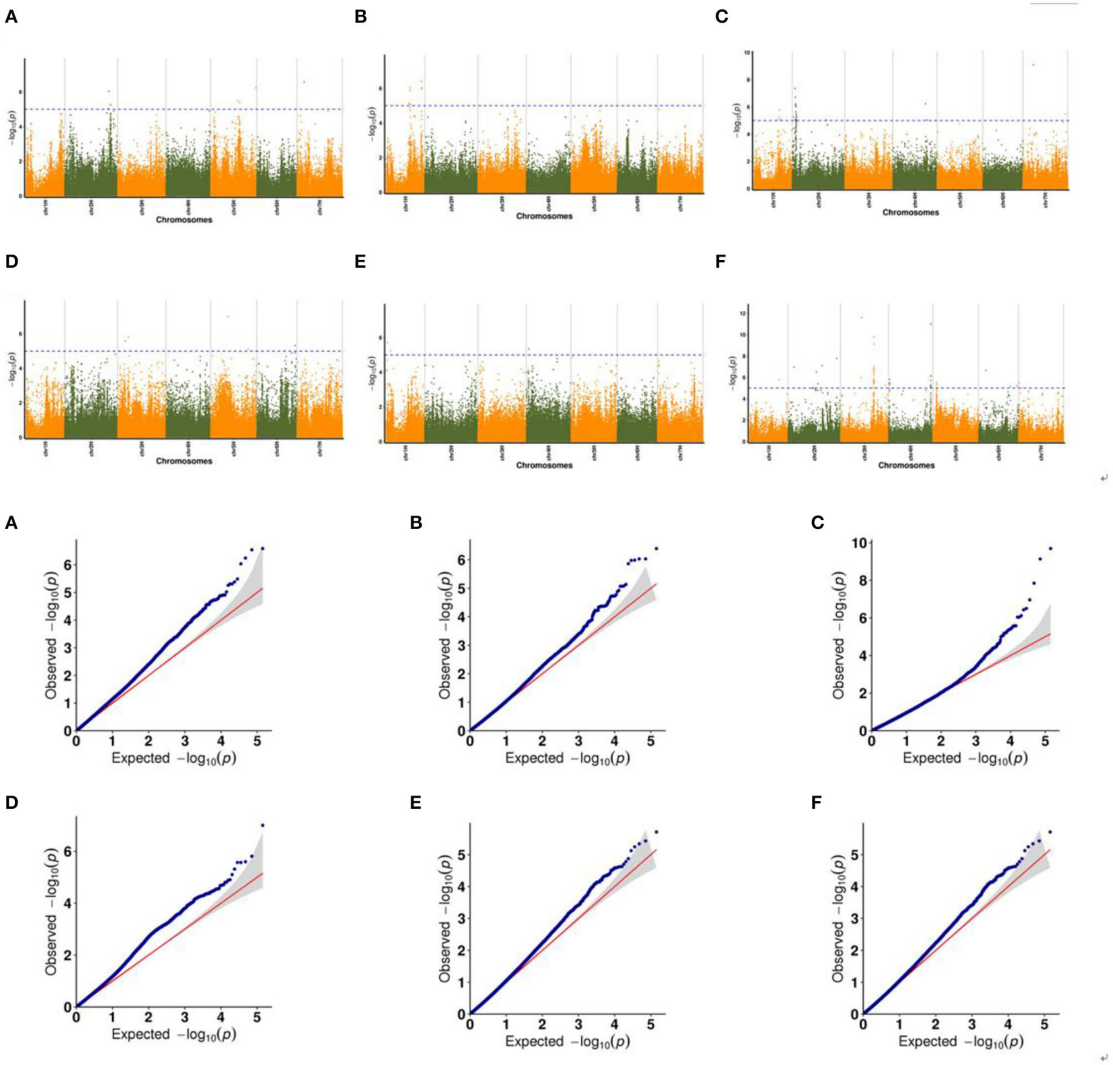
Manhattan maps and Q–Q plots representing the SNP markers associated with drought resistant in multiple trials of GWAS associated with spike development. **(A,B)** ESNP, **(C)** MEN, **(D)** MSL, **(E)** RSWD, and **(F)** TGW.

In the following part, we made a detailed explanation of some phenotypes which have shared SNP markers in the three relational models. The Manhattan plots showed significant correlation between SNP markers on multiple chromosomes and traits, while the Q–Q plots showed the relationship between observed *p*-values and expected *p-*values for each SNP marker (Figure 5, Supplementary Table 2).

Figure 5 showed the association between SNP markers and effective spike number of plants (ESNP) phenotype with Manhattan map and corresponding Q–Q plot. For plants cultured in greenhouse in Xining city during 2019, two SNP markers chr7H_102929775 and chr7H_102929728 with known functions were obtained. Both of them locate on the gene Ankyrin repeat domain-containing protein (*EMB506*). Gene *EMB506* is expressed at flowering and heading stage and closely associated with the character (spike number) (Despres et al., 2001), so it is likely to be the effector gene. There were another two SNP markers detected in plants cultured in filed in Menyuan city during 2019. One was chr1H_12690373, located on the Protein Dicer (*DCR*) gene, which is required for cutin polyester formation (Panikashvili et al., 2009). The other was chr1H_512690373, located on *APD2* gene involved in male gametophyte development (Luo et al., 2012). For main spike number (MEN) phenotype, 23 SNP markers were detected on chromosomes 1H, 2H, and 7H in plants which were cultured in filed in Xining city during 2019. On chromosome 2H, 18 SNP markers were found, including one aldehyde reductase gene Neuroplastin (*SDR1*), one gene interrelated with chloroplast development and plant growth named Probable GTP-binding protein (*OBGC1*), and three bacterial infection related genes Peroxidase 2 (*PRX112*), Probable acyl-CoA dehydrogenase (*IBR3*) and Ethylene-responsive transcription factor (*RAP2*-3). The ABC transporter G family member 11 (*ABCG11*) gene located on chromosome 2H was highly expressed in flowers and young seeds and was closely related to spike number (Panikashvili et al., 2010) so that *ABCG11* was likely to be the effector gene of the trait. Our results showed that two SNP markers (chr3H_152206655 and chr5H_250095923) connected with main spike length (MSL) phenotype. Thereinto, ATP-dependent Clp protease proteolytic subunit-related protein 1 (*CLPR*) is considered to regulate chloroplast and plant development. Deletion of *CLPR* alleles resulted in embryonic development delay and leaf albinism (Kim et al., 2009). Root and shoot dry weight (RSWD) phenotype was represented by chr1H_64014764 on Ypt Interacting Protein 4b (*YIP4B*) gene. The *YIP4B* regulates cell wall composition and participate in root and hypocotyl elongation (Gendre et al., 2013). As for thousand grain weight (TGW) phenotype, the first SNP peak was found at chr3H_482958549, and mapped to photosynthesis related gene d-glycerate 3-kinase, chloroplastic (*GLYK*). The second SNP peak was found at chr3H_489630701, and mapped to iron accumulation associated gene Geranylgeranyl pyrophosphate synthase (*BTS*).

## Discussion

Hulless barley is rich in nutrients and is the main food source for Tibetan people (Bonoli et al., 2004; Siebenhandl et al., 2007; Kohyama et al., 2008; Zhao et al., 2015). It grows on the Qinghai–Tibet Plateau and is the only crop that can grow at high altitude of 4,200–4,500 m (Dai et al., 2012; Zhong et al., 2016). However, at present, with the aggravation of drought on the Qinghai–Tibet Plateau (Meng et al., 2017), the selection of drought-tolerant hulless barley strains has become an urgent affair. However, the genetic resource that could be used to assist hulless barley molecular breeding was scarce. In this study, GWAS was used to map SNP markers related to drought tolerance in hulless barley. The SNP markers identified in this study will be used to analyze drought tolerance of hulless barley and facilitate the selection of drought-tolerant strains.

In this study, 269 lines of hulless barley were selected for drought treatment under laboratory and field conditions. Significant phenotypic variation in effective spike number of plants (ESNP), main spike number (MEN), main spike length (MSL), root and shoot dry weight (RSWD), and thousand grain weight (TGW) have been identified under drought conditions. These results indicated that the selected lines could play an important role in exploring the drought-tolerance genes of hulless barley. Hulless barley has great plasticity in adapting to drought stress, which will provide reference for the breeding process of superior hulless barley strains and improve the drought tolerance of hulless barley.

Using the hulless barley GWAS panel, 29 SNPs loci and five candidate genes connected with all spike traits (including ESNP MEN and MSL) were identified. As for ENSP, markers distributed on chromosomes 7H and chromosomes 1H were correlated, a total of 4 SNPs loci on three genes (*EMB506, DCR*, and *APD2*) were identified. *ABCG11* and *CLPR2* are two effector genes for MEN and MSL traits, respectively. Among the genes related to spike traits, *DCR* and *ABCG11* plays a key role in cuticle formation (Panikashvili et al., 2010; Rani et al., 2010). As the contact zone between the plant and the environment, cuticle has been well-characterized for its multiple roles in the regulation of gas exchange, epidermal permeability, and non-stomatal water loss (Sieber et al., 2000). So, it is not surprising that *dcr* mutants show increased water loss and increased sensitivity to drought conditions (Panikashvili et al., 2009). Besides, *EMB506* and *CLPR2* genes are associated with chloroplast and plant growth (Despres et al., 2001; Rudella et al., 2006). The lack of *CLPR2* gene causes leaf albinism and undoubtedly affects photosynthetic efficiency and crop yield (Kim et al., 2009). For RSWD at the dehydrated growth condition, *YIP4B* gene represented by chr1H_64014764 SNP was identified. In *Arabidopsis thaliana, YIP4B* affects root and hypocotyl growth through elongation rather than cell division (Gendre et al., 2013). Two genes located on Chr3H were identified for TGW trait. Among them, *GLYK* catalyzes the termination of the C2 cycle in photosynthesis, which is an indispensable auxiliary metabolic pathway for the C3 cycle of photosynthesis. The presence of this gene ensures the normal growth of terrestrial plants in an oxygen-containing atmosphere and avoids photoinhibition (Boldt et al., 2005). As iron sensors, *BTS* gene plays a vital role in modulating iron homeostasis (Zhang et al., 2015).

The mutation of these loci under drought conditions and the resulting phenotypic changes undisputedly gives us huge inspiration. Further development of these SNPs and genes will provide new insights into improving crop phenotypic traits and make plants develop in an environment-adapted direction. For instance, drought-tolerant, higher-yielding plants could be created by genetically modifying these loci. These findings will simplify the tedious process of hybridization and culture, and turn to use molecular methods for seedling breeding, which will reduce our experimental time greatly. What is more exciting is that it also provides direction for drought-tolerance selection of other economic crops besides hulless barley.

In summary, we used 5,532,468 SNP markers from 269 hulless barley lines to analyze the association between phenotypic values and genotypes of drought-tolerance traits in this study. The SNP markers association with spike traits (chr7H_102929775, chr7H_102929728, chr1H_512690373, and chr1H_512690373, chr1H_349621827, chr1H_349622062, chr2H_39562071, chr2H_47246481, chr2H_47623192, chr2H_47623303, chr2H_48148956, chr2H_48534579, chr2H_48534763, chr2H_48796138, chr2H_496935399, chr2H_496935424, chr2H_54436239, chr2H_54436346, chr2H_55411310, chr2H_55768675, chr2H_57397060, chr2H_57539586, chr2H_62893696, chr2H_64170225, chr7H_149587366, chr7H_158720411, chr7H_621337028, chr3H_152206655, and chr5H_250095923), RSWD trait (chr1H_64014764), and TGW trait (chr3H_482958549, chr3H_489630701) were identified. Under drought conditions, the mutation of these SNPs loci possibly lead to phenotypic changes and improve the adaptation of hulless barley to drought environment. In conclusion, the SNPs identified in this study can be used in drought-tolerance gene analysis, and can provide valuable information for further improvement of crop yield, quality, and adaptability.

## Data Availability Statement

All the raw genome data can be found in the National Genomics Data Center (NGDC) BioProject database with the accession number PRJCA009869.

## Author Contributions

ZF and KW contributed to the conceptualization and methodology. JL contributed to the experimentation, data analysis, and first draft preparation. XY, YY, JL, and LA contributed to the experimentation and data analysis. JL, ZF, and KW contributed to the supervision and manuscript editing. All authors have read and agreed to the published version of the manuscript.

## Funding

This work was supported by funds from the National Key R & D Program of China (2018YFD1000705 and 2018YFD1000700), National Natural science Foundation of China (32060480), the China Agriculture Research System (CARS-05), the International Science and Technology Cooperation in Sichuan Province of China (Grant No. 2021YFH0113), Qinghai Provincial Academy of Agriculture and Forestry Innovation Fund (2018-NKY-12), and the Double Support Program for Discipline Construction of Sichuan Agricultural University in China.

## Conflict of Interest

The authors declare that the research was conducted in the absence of any commercial or financial relationships that could be construed as a potential conflict of interest.

## Publisher's Note

All claims expressed in this article are solely those of the authors and do not necessarily represent those of their affiliated organizations, or those of the publisher, the editors and the reviewers. Any product that may be evaluated in this article, or claim that may be made by its manufacturer, is not guaranteed or endorsed by the publisher.
